# Analysis of Musculoskeletal Biomechanics of Lower Limbs of Drivers in Pedal-Operation States

**DOI:** 10.3390/s23218897

**Published:** 2023-11-01

**Authors:** Song Zhang, Hailin Kui, Xiangyu Liu, Zhonglin Zhang

**Affiliations:** 1 Department of Automotive Engineering, Hebei Jiaotong Vocational and Technical College, Shijiazhuang 050035, China; z954509395@163.com; 2 Transportation College, Jilin University, Changchun 130022, China; 3 College of Biological and Agricultural Engineering, Jilin University, Changchun 130022, China; 4 College of Mechanical and Electrical Engineering, Harbin Engineering University, Harbin 150001, China

**Keywords:** vehicle ergonomics, musculoskeletal biomechanics, pedal operation, car seat heights, electromyography (EMG), musculoskeletal model, OpenSim

## Abstract

In this study, to establish the biomechanical characteristics of commercial vehicle drivers’ muscles and bones while operating the three pedals, a driver pedal-operation simulator was built, and the real-life situation was reconstructed in OpenSim 3.3 software. We set up three seat heights to investigate the drivers’ lower limbs, and the research proceeded in two parts: experiment and simulation. Chinese adult males in the 95th percentile were selected as the research participants. In the experiment, Delsys wireless surface electromyography (EMG) sensors were used to collect the EMG signals of the four main muscle groups of the lower limbs when the drivers operated the three pedals. Then, we analyzed the muscle activation and the degree of muscle fatigue. The simulation was based on OpenSim software to analyze the driver’s lower limb joint angles and joint torque. The results show that the activation of the hamstrings, gastrocnemius, and rectus femoris muscles were higher in the four muscle groups. In respect of torque, in most cases, hip joint torque > knee joint torque > ankle joint torque. The knee joint angles were the largest, and the ankle joint angles changed the most. The experimental results provide a reference for improving drivers’ handling comfort in commercial vehicles and provide theoretical bases for cab design and layout optimization.

## 1. Introduction

Commercial vehicle drivers often drive for extended periods, and as car seats and pedals are some of the components with which drivers make direct contact, the design of these affects drivers’ handling comfort [1,2]. Reasonable human–computer interaction design can effectively improve driving comfort. Traditional automobile manufacturers conduct driver comfort research based on subjective experiences, historical data, and costly prototype experiments [3]. Due to the limitations of traditional methods, computer-aided engineering (CAE) has been introduced into automotive ergonomics design, such as RAMSIS in Germany, ERGO and JACK in the United States, and Pam Comfort in France [4]. CAE technology enables automobile manufacturers to focus on drivers’ driving posture, the accessibility of cockpit components, and drivers’ field of vision through a 3D human–vehicle model at the beginning of the design process to ensure better ergonomic design. Due to the inability of CAE calculation methods to reveal the fatigue mechanisms of drivers in a more detailed manner at the level of human bones and muscles, it is difficult to quantitatively describe and simulate changes in the biomechanical characteristics of drivers’ bones and muscles. Many human musculoskeletal models have been established based on the combination of biomechanics and ergonomics disciplines [5].

In recent years, scholars have used computers to establish musculoskeletal models for biomechanical simulation analysis [6,7], commonly using software such as OpenSim [8,9], Anybody [10], and Msms. Christopher et al. [11] first established a lumbar musculoskeletal model based on the OpenSim platform. Actis et al. [12] researched lumbar and lower limb loads by comparing the simulation data and EMG signal data of normal individuals and unilateral amputation patients combined with the OpenSim musculoskeletal model. Aliah et al. [13] explored the effects of seat height, seat inclination, and accelerator pedal spring stiffness on drivers’ muscles and spine when driving on urban roads based on a human musculoskeletal model. They proposed optimal values for seat and pedal parameters. Grujicic et al. [6] established a musculoskeletal model of a car seat and human body. They studied the influence of human–seat interaction and human–machine seat design on driver comfort.

The above models effectively reveal the seat and pedal effects on the driver’s musculoskeletal system. However, previous studies have focused more on the driver’s single seat height and single pedal operation without further considering the biomechanical characteristics of the drivers when operating different pedals at different seat heights. For this reason, we used the international mainstream biomechanical analysis software OpenSim to establish a musculoskeletal mechanics model for drivers operating different pedals. Dynamic changes in the lower limb joint angles and joint torque of drivers were analyzed by adjusting seat height, which provides a detailed reference for driver seat and pedal design. We also introduced electromyography (EMG) for comfort evaluation, which can more comprehensively characterize the fatigue state of drivers.

Regarding comfort evaluation, this usually includes subjective and objective evaluation [14]. Subjective evaluation refers to using various comfort scales and survey questionnaires [15], but individual factors greatly influence this method and introduce uncertainty [16]. In order to make up for any relevant shortcomings, objective evaluation is introduced, such as electromyography, electroencephalography, and electrocardiography [17]. Since the muscles, bones, and joints of the human body constitute the whole motor system, the driver’s operation of the vehicle is driven by the cooperation of these elements, which are closely related. An EMG signal can objectively reveal muscle activation [18], so EMG is introduced to more comprehensively reveal drivers’ biomechanical characteristics.

As nonlinear and non-stationary signals, EMG signals have a frequency range of 20–500 Hz, with the main energy concentrated between 50 and 150 Hz [19]. However, within this frequency range, there are many noise signals unrelated to EMG signals, such as motion artifact noise caused by the electrical properties of the skin–electrode interface [20,21], the inherent noise of electronic components included in electronic devices, and power frequency noise caused by mains and instrument circuits [22]. The noise of motion artifacts and electronic components is generally in the range of 0–60 Hz, and the power frequency noise is 50 Hz. The above noises will reduce the quality of EMG signals, thereby affecting the calculation of muscle activation. Therefore, bandpass filtering and 50 Hz notch filtering are used for preprocessing in the analysis of EMG signals to obtain a pure signal and improve signal quality.

Jae-Jun Kim [23], taking bus drivers as the object, combined with the median frequency of EMG, evaluated the fatigue status of muscle groups of lower limbs during pedal operation. Wang et al. [24], based on the root-mean-square of forearm muscle EMG, analyzed the effect of an adaptive shared steering control system on driver distraction behavior. Zhao et al. [21] used OpenSim to determine the main muscles that drive lower limb movement and established a prediction model of lower limb joint angles and torque based on the EMG signals of these muscles.

When a person is driving, the driver’s main stress areas are the muscles of the waist, chest, and legs that come into contact with the seat. Pedal operation requires repeated use of the legs and feet, causing a burden and fatigue on the lower limbs. The existing literature provides very little information on fatigue in respect of the leg muscles during prolonged car driving [25]. In this research, we studied the joints of the legs and the muscles that primarily generate force when operating pedals.

1. In summary, most existing studies have analyzed the impact of a single seat height and single pedal manipulation on the biomechanical characteristics of drivers, and there is little research on the lower limbs of drivers. There is also a lack of quantitative analysis of the impact of different pedal types and seat heights on the comfort of driver lower limb manipulation. Based on this, we established a pedal-manipulation simulator to collect the EMG signals of the main muscles in the driver’s lower limbs and analyzed the muscle activation. We also used OpenSim software to reconstruct the model and analyzed the driver’s joint angle and joint torque. This paper provides insights into the changing rules of musculoskeletal biomechanics and EMG signals for the precise design of vehicle seats and the three driving pedals, as well as quantitative design bases for seat-height adjustments. A simulated driving platform was built using the Vicon infrared camera system to conduct three-pedal operation experiments for drivers in normal vehicle conditions. We selected three groups of different commercial vehicle seat heights. Through the driving simulator, we obtained the kinematics (marker-point 3D coordinates), dynamics (external 3D force), and EMG data of drivers’ lower limbs.
2. We performed noise reduction, rectification, and linearization analysis of the driver’s lower limb EMG data collected during the experiment, analyzed the driver’s leg muscle activation, and thus analyzed the degree of muscle fatigue. Muscle activation is the ratio of muscle electrical signals under the operation state to those under the maximum force state. When muscle activation is low, it indicates a higher level of driver comfort.
3. The driving situation was reconstructed based on OpenSim software. The collected kinematic data (marker-point 3D coordinates) could be used to drive the OpenSim model and reproduce the driver’s operational actions. The reverse kinematics analysis using OpenSim software could analyze the angle of the driver’s lower limb joints. Furthermore, based on inverse kinematics, the collected dynamic data (external 3D force) were used for inverse dynamics analysis to analyze the joint torque of the driver’s lower limbs. Combined with muscle activation, changes in the biomechanical characteristics of the lower limbs when the driver operated the pedals were analyzed.

This paper can provide insights into the changing rules of musculoskeletal biomechanics and EMG signals for the precise design of vehicle seats and the three driving pedals, as well as quantitative design bases for seat-height adjustments.

## 2. Design of Lower-Limb Data Collection Experiment

### 2.1. Experimental Equipment

#### 2.1.1. Three-Dimensional Motion-Capture System

**Figure 1:**
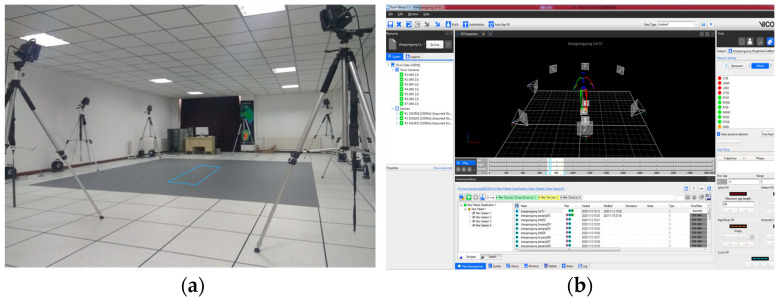
Vicon infrared three-dimensional motion-capture system. (**a**) Actual situation; (**b**) situation displayed by Nexus software.

In this experiment, a Vicon infrared three-dimensional motion-capture system was used to acquire real-time kinematic data of the driver during the operation process. The system included seven infrared cameras with a sampling frequency of 100 Hz, and the cameras were evenly distributed to ensure that all markers could be identified, as shown in Figure 1a. Figure 1b shows the offline data-processing software Nexus 2.5 corresponding to Vicon.

#### 2.1.2. Surface EMG Acquisition System

**Figure 2:**
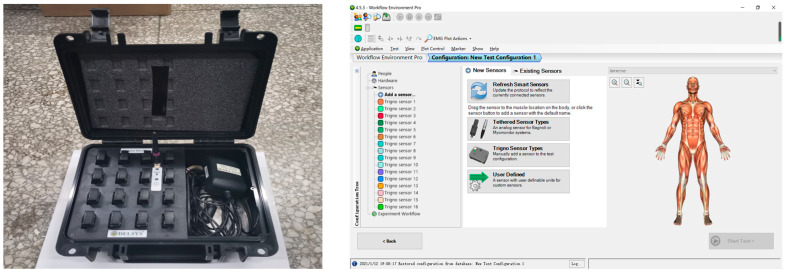
Trigno Mobile wireless surface electromyography system.

We used a Delsys Trigno Mobile wireless surface electromyography sensor from the United States, as shown in Figure 2 below, which has 16 channels and can simultaneously collect electrical signals from multiple groups of muscles at a sampling frequency of 2000 Hz. Using this device, the collected electromyography data can be exported and stored in real time, facilitating the subsequent calculation of muscle activation.

#### 2.1.3. Driving Simulation Platform

The aim of the experiment was to establish a pedal-operation simulation platform for studying drivers’ biomechanical characteristics.

SAE J1516 proposes a method for calculating pedal inclination based on H-point heights (H30). The formula is as follows:

A47 = 78.96 − 0.15(H30) − 0.0173(H30)^2^, (1)

where A47 is the pedal angle (°) and the unit of H30 is cm.

The pedal center heights can also be calculated through the pedal inclination angles. Assuming that the distance from the accelerator pedal heel point (AHP) to the ball of the foot (BOF) is L (SAE recommends that L be 200 mm), the pedal center height formula is as follows.

Z_AP_ = Lsin(A47). (2)

The seat heights (H30) were set to 405 mm, 455 mm, and 505 mm, and the layout parameters of the driving simulator are shown in Table 1.

**Figure 3:**
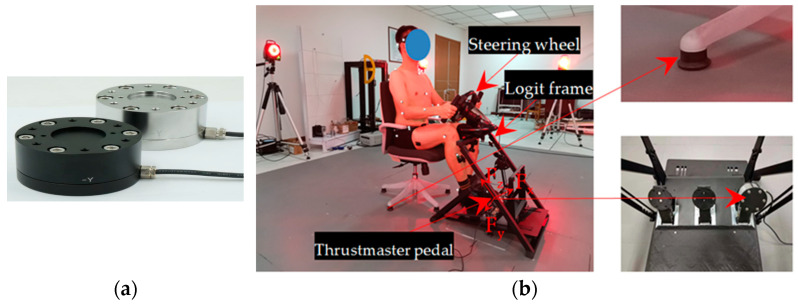
Pedal-operation experiment. (**a**) LH-SZ-6W sensors were used to collect plantar-force data; (**b**) pedal-operation simulator.

Figure 3a shows the LH-SZ-6W three-dimensional force sensor fixed on the pedal using a 3D-printed fixture during the experiment. Figure 3b shows the pedal-operation simulator with a 3D-printed steering wheel and a diameter of 450 mm. The frame is a Logitech g29 racing simulator, in which the steering wheel height, steering wheel inclination angle, pedal center height, etc. can be adjusted. The pedal is a Thrustmaster racing pedal, a floor-mounted pedal with adjustable pedal inclination and feedback force. The feedback forces of the accelerator, brake, and clutch pedals were set to 40 N, 120 N, and 50 N, respectively, with a distance of 70 mm between two adjacent pedals [26]. The seat can be adjusted to a height of 405–505 mm, with a backrest angle of 8–18° and a vertical H-point rise of 0°.

### 2.2. Experiment Participants

Since the structural layout design of the cab should meet the driving needs of the vast majority of passengers, 95th-percentile males were selected as the research participants based on the data provided in GB10000-88 “Human Body Size of Chinese Adults”, that is, a height of 177.5 cm and a weight of 75 kg. Three participants were selected for the EMG signal experiments, as shown in Table 2. The OpenSim simulation selected Participant 3 to calculate the joint angles and joint torque.

We measured the body dimensions of the participants before attaching the marker points, which were important factors in determining comfort and essential for constructing skeleton models in the Nexus software. As shown in Table 3, we entered these values in the “properties” toolbar of the Nexus software after the experiment started. At the same time, based on the principle of basic consistency between the left and right sides of the human body, we measured the data of the drivers’ right sides and filled in the same values on the body data panel of the left sides.

#### 2.2.1. Muscle Selection and Sensor Pasting

Research has shown that in the process of pedal operation, the main muscles that generate force and exhibit apparent changes are the rectus femoris, hamstring, gastrocnemius, and tibialis anterior muscles [27]. The hamstring includes the semimembranosus, the semitendinosus, and the long head of the biceps femoris. The gastrocnemius includes the medial and lateral head of the gastrocnemius muscle. This study was conducted in respect of four muscles: the rectus femoris, the long head of the biceps femoris in the hamstring muscle group, the medial head of the gastrocnemius in the gastrocnemius muscle group, and the anterior tibial muscle.

**Figure 4:**
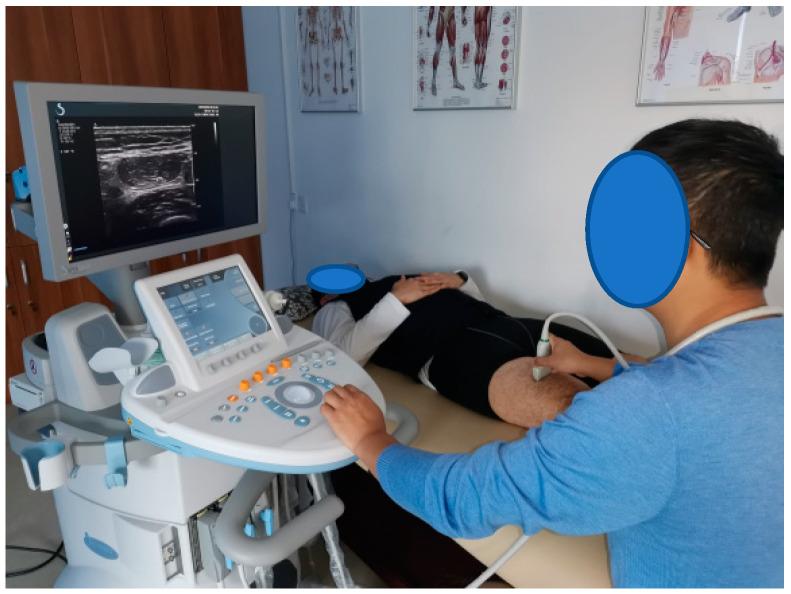
Aixplorer ultrasound imaging system.

**Figure 5:**
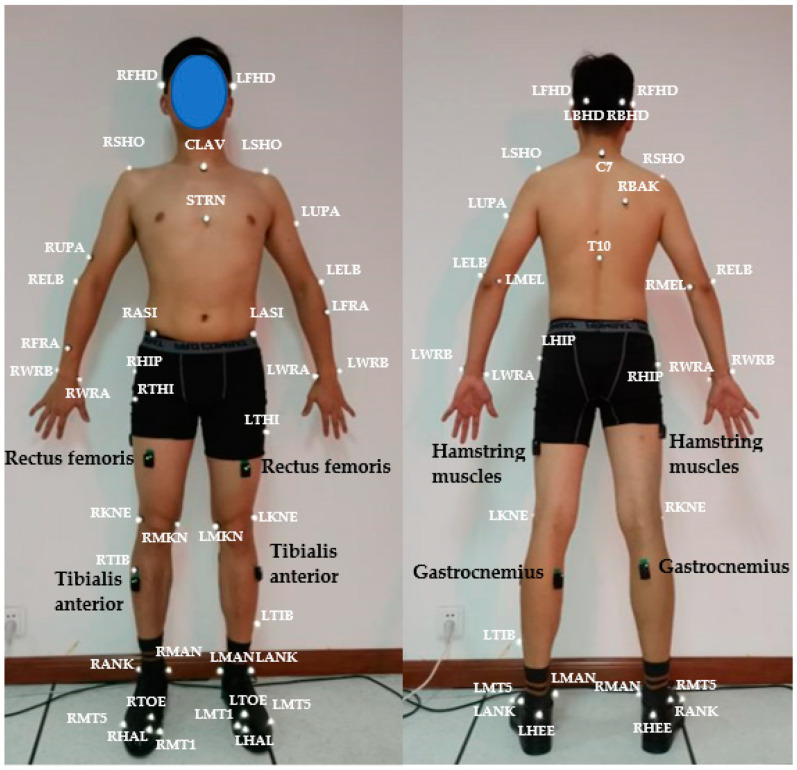
Marker points and EMG sensor pasting.

After the participants had relaxed completely, an Aixplorer high-frequency ultrasound imaging system was used to determine the muscle positions of the rectus femoris, hamstring (long head of biceps femoris), tibialis anterior, and gastrocnemius muscles (medial head of gastrocnemius). Figure 4 shows muscle localization using the ultrasound imaging system. The muscle position is shown in Figure 5.

#### 2.2.2. Maximum Voluntary Contraction (MVC) Calibration

Muscle activation can be represented by the ratio of the current EMG signal to the maximum voluntary contraction state EMG signal. After preparation, we conducted MVC calibration experiments on all four muscle groups to collect the EMG signal of each muscle at its maximum output [27,28]. Each MVC position lasts for 3–4 s. To avoid muscular fatigue, a 2 min rest was allowed between MVC experiments [29].

- Rectus femoris: The participant was sitting, maintaining the knee joint at 90°. The collector applied resistance at the knee joint to prevent the thigh from lifting.
- Hamstring muscle: The participant lay prone on the platform, raised the lower leg to maintain an angle of 20–30 degrees with the ground, bent the knee vigorously, and applied resistance at the ankle.
- Tibial anterior: The participant was sitting, maintaining the knee joint at 90°, and the collector applied resistance on the foot surface to prevent the foot from lifting.
- Gastrocnemius: The participant was sitting, maintaining the knee joint at 90°. The collector applied resistance at the heel to prevent the heel from lifting.

#### 2.2.3. Motion-Capture System Markers

Since this experiment needed to reflect the motion state of the driver’s whole body and obtain kinematics data for the whole body, it was necessary to establish a reflective marker system of the subject’s whole body. We referred to the commonly used marker-point system to drive the selection of a fifteen-link multi-rigid body model. The selected marker-point system has 49 marker points, as shown in Figure 5. 

#### 2.2.4. Scheme Design and Data Collection

The experimental steps were as follows:

1. Seat height was adjusted to H30 = 405 mm, corresponding to pedal inclination and pedal center height. The specific parameters are shown in Table 1 above. The experimenters adjusted themselves to a comfortable driver layout and sitting position.
2. After hearing the experimenter’s starting command, the participant grasped the steering wheel with both hands, placed the right foot on the accelerator pedal but did not push down, and placed the left foot on the floor for 3 s. The above actions were repeated three times, with the subject resting for over 1 min after each action. The experimenter took the average of three actions as the final calculation value. The experimenter collected the EMG signal, kinematics, and dynamics data of the lower limbs when the pedal was not pushed down and then saved them.
3. The right foot movements of the subject were changed to three sets of actions: pressing the accelerator pedal to half the stroke at a constant speed, pressing to the bottom at a constant speed, releasing the pedal at a constant speed, and placing the right foot on the pedal. Each pedal operating position lasts for 3–4 s. The above two sets of steps were repeated.
4. Seat height was adjusted to 455 mm and then 505 mm. The above three steps were repeated.
5. The pedals were changed to the brake pedal and clutch pedal. The above four steps were repeated.
6. A new subject repeated the above five steps.

The comfort experiment in respect of lower limb pedal operation for commercial vehicle drivers with different seat heights is shown in Figure 3b above.

## 3. Results and Discussion

### 3.1. Analysis of Activation of Lower Limb Muscle Groups

#### 3.1.1. Calculation of Muscle Activation

After collecting the drivers’ EMG signal data, the data were subjected to 25–250 Hz bandpass filtering and 50 Hz notch filtering to remove noise from the experiment. Then, the signal was subjected to full-wave rectification and low-pass filtering. We calculated the driver’s muscle activation based on the EMG data collected from the MVC calibration experiment [30]. The formula for calculating muscle activation is

**Figure 6:**
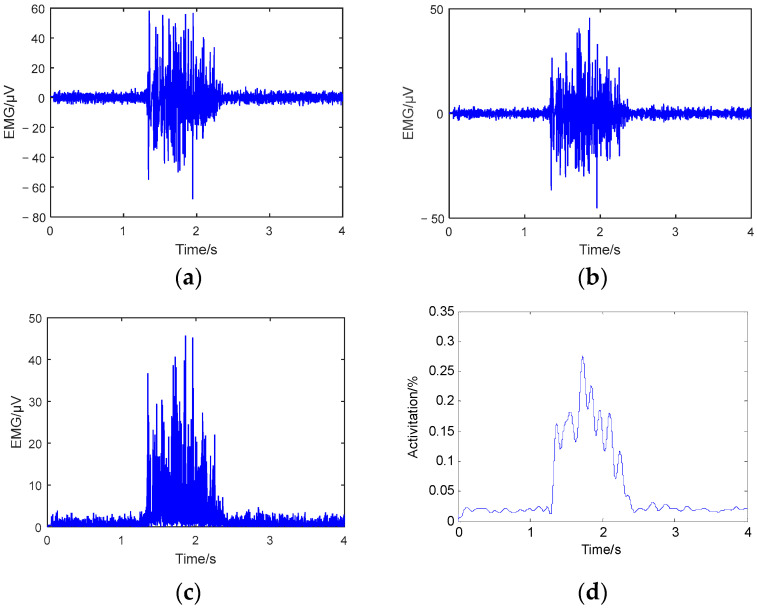
Calculation of muscle activation: (**a**) original EMG signal, (**b**) denoised EMG signal, (**c**) rectified EMG signal, and (**d**) muscle activation.

A_0_ = EMG/EMG_max_, (3)

where A_0_ represents muscle activation, which is a dimensionless value. EMG represents the electrical signal generated by muscle force in the current state. EMG_max_ represents the electrical signal generated by the muscle when it is in the maximum state of force, and we used MVC action calibration to obtain the parameter. The calculation process of muscle activation is shown in Figure 6 [30,31]. When muscle activation is low, it means that the muscles are more relaxed, and the comfort level is higher [27].

#### 3.1.2. Calculation of Muscle Activation during Pedal Operation

**Figure 7:**
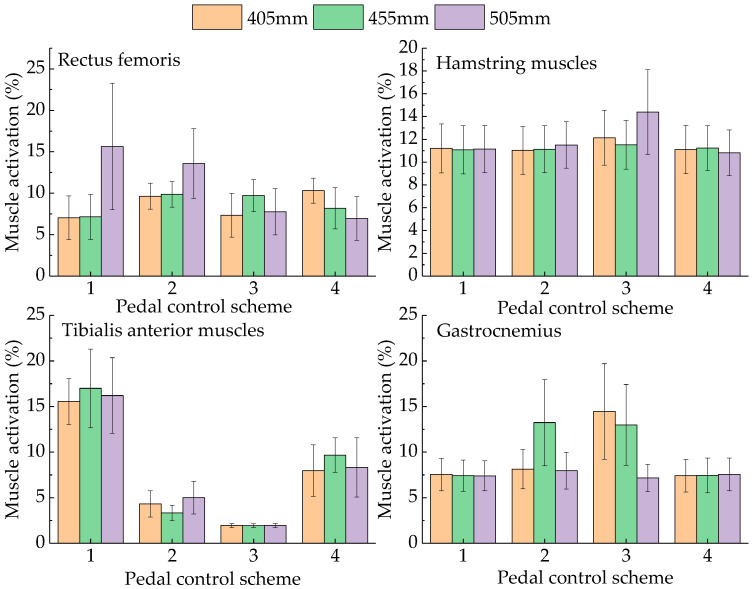
Muscle activation occurring when the drivers operated the accelerator pedal.

In this study, four experimental schemes were designed for pedal operation. In Scheme 1, the driver lightly put their foot on the pedal. In Scheme 2, the driver pressed the pedal at a constant speed to half the stroke. In Scheme 3, the driver pressed the pedal to the bottom at a constant speed. In Scheme 4, the driver relaxed the pedal at a constant speed until the moment before their foot left the pedal. We calculated the mean and standard deviation of muscle activation during pedal operation for three participants. The muscle activation when the drivers operated the accelerator pedal at seat heights of 405 mm, 455 mm, and 505 mm is shown in Figure 7.

As shown in Figure 7, during the process of pressing down the accelerator pedal, when the seat heights are 405 mm and 455 mm, the activation of the rectus femoris increases first and then decreases. When the seat height is 505 mm, the activation of the rectus femoris is the largest when not pressed down and then decreases gradually as the stroke progresses. The activation of the hamstring muscle first decreases and then increases when the seat height is 405 mm and gradually increases when the seat height is 455 mm and 505 mm. The activation of the tibialis anterior muscle continuously decreases when pressing down the pedal. When the seat height is 405 mm, the activation of the gastrocnemius gradually increases. When the seat height is 455 mm and 505 mm, the activation of the gastrocnemius first increases and then decreases. When the pedal is depressed, the gastrocnemius, tibialis anterior, and rectus femoris have the largest range of change, and the maximum changes are 24.62%, 14.5%, and 13.2%, respectively, indicating that the gastrocnemius plays an essential role in operating the accelerator pedal [25]. 

The above four muscle groups are the main contributing muscle groups of the drivers’ lower limbs. We gave them the same weight and accumulated the muscle activation of the four muscle groups in the complete operation process under the same seat height. The activation of the lower limbs for a seat height of 405 mm, 455 mm, and 505 mm was 216.13%, 226.53%, and 222.71%, respectively, indicating that the driver’s lower limb comfort was the highest when the seat height was 405 mm.

**Figure 8:**
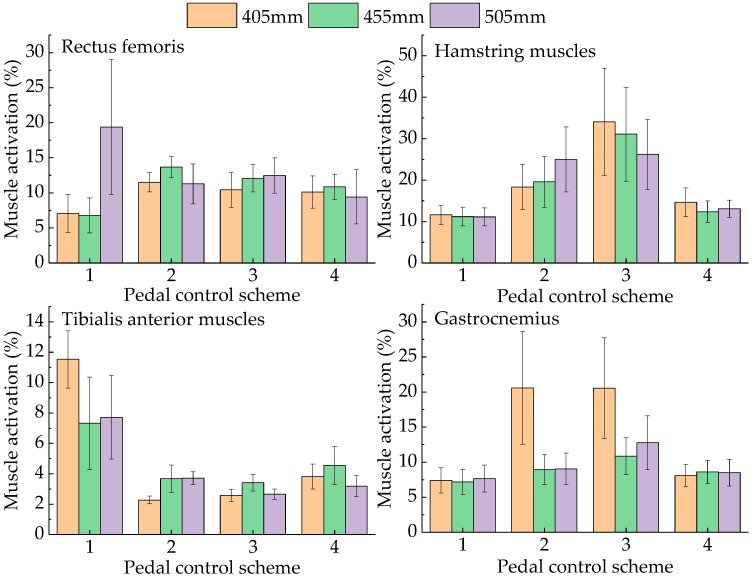
Muscle activation occurring when the drivers operated the brake pedal.

As shown in Figure 8, during the process of pressing down the brake pedal, when the seat height is 405 mm and 455 mm, the activation of the rectus femoris increases first and then decreases, which is the same result as that of pressing the accelerator pedal. When the seat height is 505 mm, the rectus femoris is the largest when the pedal is not pressed, and the activation first decreases and then increases with the progress of the stroke. The activation of the hamstring muscles gradually increases, similar to the change when the accelerator pedal is pressed. The activation of the tibialis anterior muscle gradually decreases at seat heights of 455 mm and 505 mm, but at 405 mm, it decreases first and then increases. The activation of the gastrocnemius first increases and then decreases at a seat height of 405 mm, and gradually increases at 455 mm and 505 mm. The activation of the hamstrings and rectus femoris is increased compared with that of the accelerator pedal. The reason for this is that the brake pedal resistance is large at this time [26], which requires the leg to generate more operation force.

Using the same procedure used for the accelerator pedal, the activation of the four groups of muscles during complete operation at the same seat height was accumulated. The activation of the lower limbs corresponding to the seat height from small to large (405 mm, 455 mm, and 505 mm) was 314.84%, 275.44%, and 286.41%, respectively, indicating that the driver’s lower limb comfort was the highest when the seat height was 455 mm.

**Figure 9:**
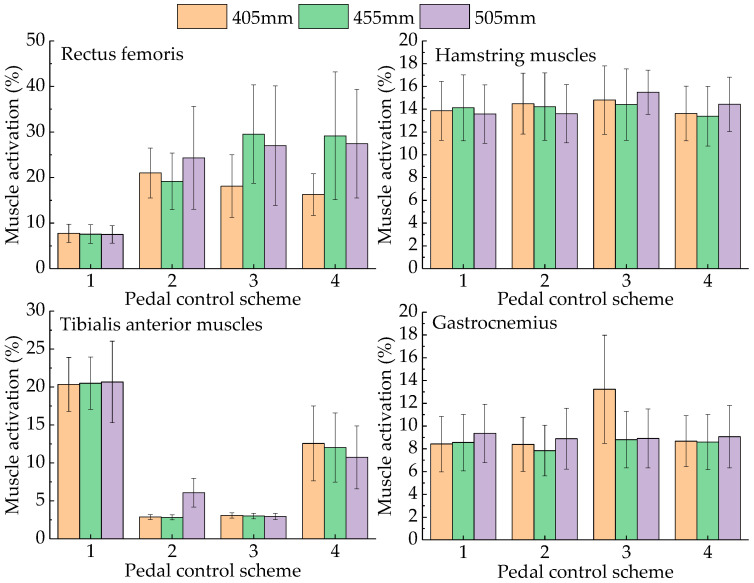
Muscle activation occurring when the drivers operated the clutch pedal.

As shown in Figure 9, during the process of pressing down the clutch pedal, when the seat height is 405 mm, the activation of the rectus femoris increases first and then decreases. At seat heights of 455 mm and 505 mm, the activation gradually increases when the pedal is pressed. The activation of the hamstring muscle gradually increases at all three seat heights. The activation of the anterior tibial muscle first decreases and then increases at seat heights of 405 mm and 455 mm. However, it continuously decreases at a seat height of 505 mm. The gastrocnemius activation decreases first and then increases at all three seat heights. When pressing the pedal, the tibialis anterior, rectus femoris, and hamstring muscles have the largest range of change, with magnitudes of 15.61, 13.33, and 10.64, respectively.

Using the same procedure used for the accelerator pedal, the muscle activation of the four groups of muscles during complete operation at the same seat height was accumulated. The activation of the lower limbs corresponding to the seat height from small to large (405 mm, 455 mm, and 505 mm) was 251.56%, 313.55%, 327.24%, respectively, indicating that the driver’s lower limb comfort was the highest when the seat height was 405 mm.

### 3.2. Analysis of Musculoskeletal Biomechanics Based on OpenSim

#### 3.2.1. Building Driving Posture

OpenSim software is a free software package developed by Stanford University for visualizing and analyzing human musculoskeletal systems. Based on OpenSim software, we adopted the human body musculoskeletal model proposed by Raabe et al. [32]. The model is composed of 21 segments, 30 degrees of freedom, and 324 musculotendon actuators. The model’s parameters, such as trunk muscle geometry and maximum isometric joint torque, were compared and verified with experimental data, demonstrating reliability [32].

**Figure 10:**
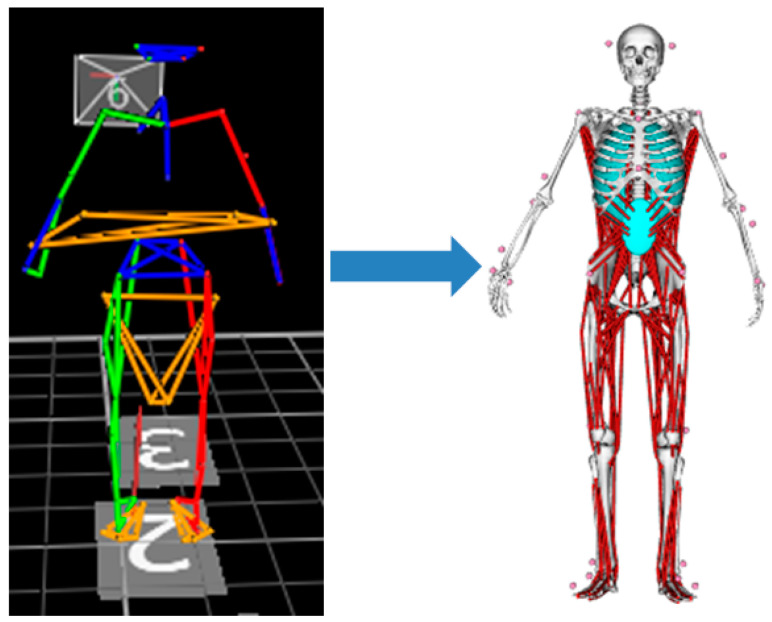
OpenSim model scaling.

As there were differences in height and weight between the selected human whole-body musculoskeletal model and the drivers selected in the experiment, the model needed to be scaled to adjust the size and quality properties of each part of the body. We used the 3D coordinates of the marker points in the static calibration actions to correspond and scale the musculoskeletal model in OpenSim, as shown in Figure 10.

**Figure 11:**
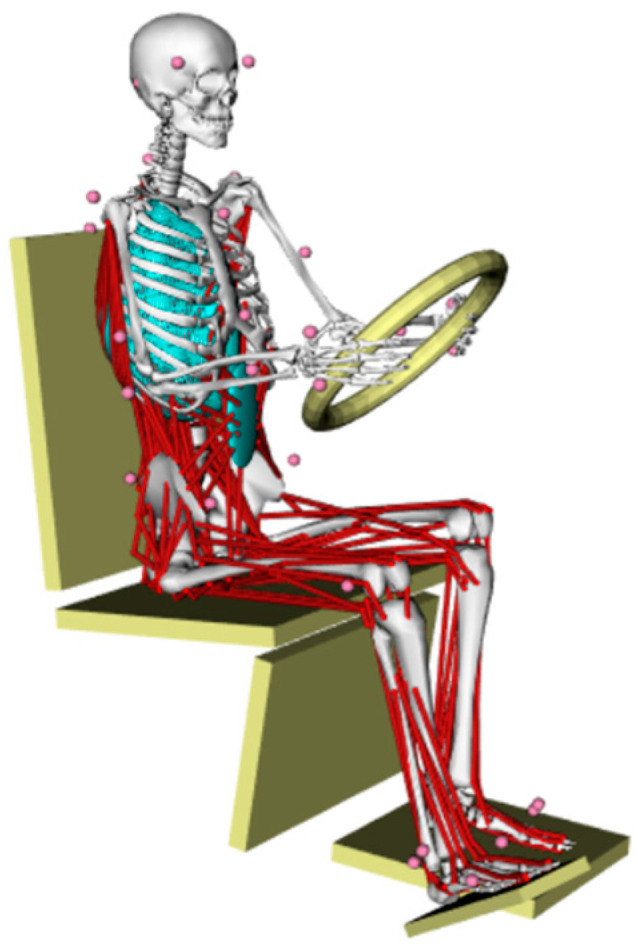
Reconstruction of driver’s operation state.

The driver pedal-operation environment model also needed a seat, steering wheel, footrest, and pedal model, so after completing model scaling, the environment model in STL format was established. After importing these models into the OpenSim software, it was necessary to apply different constraints and degrees of freedom limitations to the driver’s hands, back, and hips. In order to simulate the force condition experienced by the driver in the actual operating state, it was also necessary to add support points at the driver’s back, pelvis, femurs, left foot, and right heel according to the contact position between the driver and the driving simulator. Finally, we built the scenario as shown in Figure 11.

#### 3.2.2. Analysis of Joint Flexion Angles and Flexion Torque When Lower Limbs Operate Pedals

In the pedal-operation experiment, the complete pedal-operation process was as follows: the pedal was stationary, and then the driver pushed down on the pedal to the bottom at a constant speed. After a slight pause, the driver raised the foot to the initial position at a constant speed and stopped the foot on the pedal, finally leaving the pedal.

**Figure 12:**
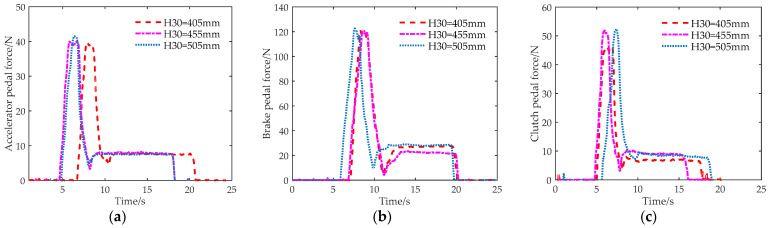
Pedal resistance at different seat heights. (**a**) Accelerator pedal, (**b**) brake pedal, and (**c**) clutch pedal.

We used the LH-SZ-6W three-dimensional force sensor to collect the corresponding pedal resistance during a complete stepping process, as shown in Figure 12. Figure 12a–c, respectively, show the relationship between the pedal force and the stepping time of the driver when pressing the accelerator pedal, brake pedal, and clutch pedal at different seat heights. Pedal force can be used as input for inverse dynamics to solve the joint torque.

The model was analyzed using inverse kinematics (IK) and inverse dynamics (ID), respectively, through the 3D coordinates of marker points and 3D forces of the LH-SZ-6W sensors. The angle and torque changes of the hip, knee, and ankle joints during the driver’s operation were inversely solved.

**Figure 13:**
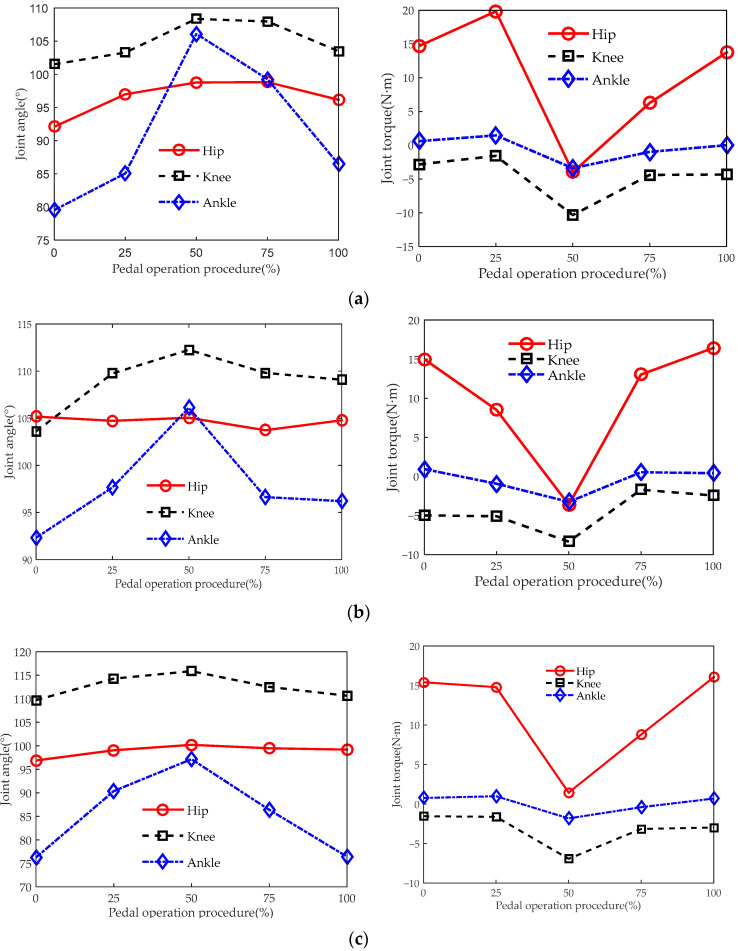
Biomechanical curves of the lower limbs when operating the acceleration pedal. The joint angles and joint torques correspond to seat heights of (**a**) 405 mm, (**b**) 455 mm, and (**c**) 505 mm.

Figure 13 reflects the lower limb joint angles and torque changes when the driver operates the accelerator pedal at different seat heights. The pedal operation process involves the driver pushing down on the pedal at a constant speed, pressing it to the bottom, and then releasing the pedal at a constant speed until the foot leaves the pedal. That is, the process of the driver pushing the pedal down is 0–50%, and the process of releasing the pedal is 50–100%.

It can be seen from Figure 13 that most joint angle changes are within the range of joint angles for a comfortable posture, that is, hip joint 95–120°, knee joint 95–135°, and ankle joint 78–105° [26]. Figure 13 shows that when stepping on the accelerator pedal at seat heights of 405 mm, 455 mm, and 505 mm, the three joint angles gradually increase in most cases. In most cases, when the driver releases the pedal, the three joint angles gradually decrease. The hip joint angle changes slightly at a seat height of 455 mm, and this is influenced by the driver’s upper-body posture. This shows that pressing the accelerator pedal can be seen as a process of lower limb relaxation, and releasing the accelerator pedal can be seen as a process of lower limb contraction. The analyses of the changing trend of joint angles are also suitable for the brake and clutch pedals.

At the beginning of contact with the pedal, due to the gravity factor of the lower limbs, each joint of the lower limbs will be subjected to the torque produced by gravity. When first touching the pedal, the lower limb joints will produce torque due to the influence of gravity of the driver’s lower limbs. Since the mass of the driver’s foot and calf is small, the knee–ankle joint produces less torque, and the hip joint produces about 15 Nm torque. 

**Figure 14:**
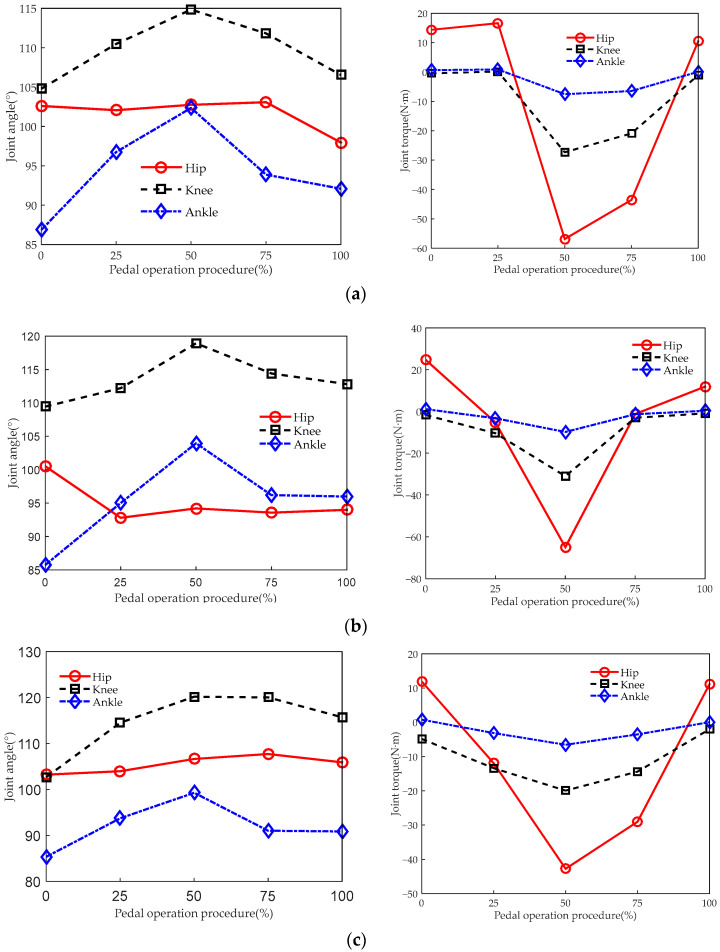
Biomechanical curves of the lower limbs when operating the brake pedal. The joint angles and joint torques correspond to seat heights of (**a**) 405 mm, (**b**) 455 mm, and (**c**) 505 mm.

**Figure 15:**
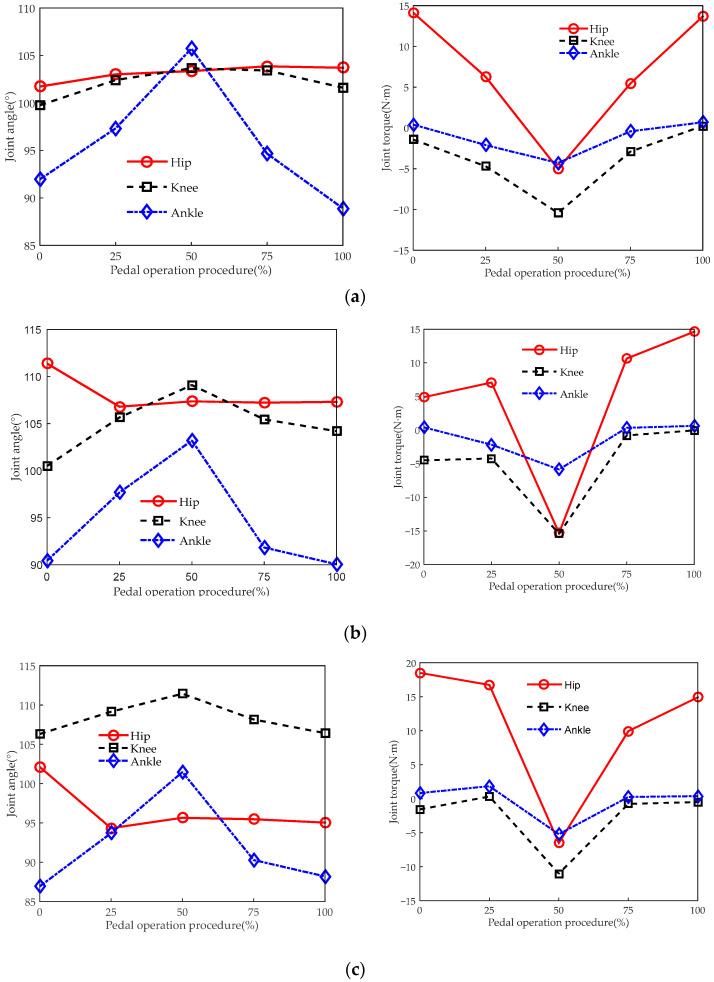
Biomechanical curves of the lower limbs when operating the clutch pedal. The joint angles and joint torques correspond to seat heights of (**a**) 405 mm, (**b**) 455 mm, and (**c**) 505 mm.

Figure 14 and Figure 15, respectively, reflect the changes in angles and torque of the lower limb joints when the driver operates the brake and clutch pedal at different seat heights.

Figure 14 and Figure 15 show that when the driver operates the brake pedal and clutch pedal, the changes in lower limb joint angles are basically the same as for the accelerator pedal and are also within the range of joint angles of comfortable posture in most cases. When operating the brake pedal, the torque of the hip and knee joints requires larger torque than when operating the accelerator pedal and clutch pedal. This is because when operating the brake pedal, compared to the accelerator and clutch pedal, the pedal resistance is larger and requires greater force from the lower limbs.

## 4. Conclusions

In this study, combined with the biomechanical characteristics of drivers’ lower limbs, an EMG experiment for multiple drivers was undertaken to analyze the changes in the drivers’ muscle activation under pedal-operation states. OpenSim simulation was used to analyze the drivers’ joint angles and torque changes under pedal-operation states.

- When operating the three pedals, the activation of the hamstrings, gastrocnemius, and rectus femoris muscles is higher in the four muscle groups. When the pedal is not pressed (that is, when the ankle joint dorsiflex movement begins), the muscle activation of the tibialis anterior muscle is maximal, and the angles and torque of the ankle joint are minimal. The activation is lower when operating the acceleration pedal, which indicates that lower pedal resistance can make the driver more comfortable. Compared with the acceleration and clutch pedals, when the brake pedal is operated, the hamstring muscle activation is obviously increased, and the hip, knee, and ankle torque is also larger. Under the three seat heights, when the driver operates the accelerator pedal and the clutch pedal, the lower limb muscle activation is lowest at 405 mm. This is because the 95th-percentile male body height and weight is larger, and the lower seat height is more comfortable. When operating the brake pedal, the muscle activation of the lower limbs first decreases and then increases with the seat height. This is because the brake pedal has the largest resistance, requiring more muscle force and force space to operate.
- The range of joint angle changes is ankle > knee > hip, which is locally different. At a seat height of 505 mm, when operating the brake pedal, the knee angle changes more than the ankle angle. Similarly, at a seat height of 505 mm, when the clutch is operated, the hip angle changes more than the knee joint angle. This is because when the seat height exceeds the optimal handling comfort height of the human body, the driver automatically adjusts their driving position to meet their comfort needs. The overall variation trend of knee and ankle torque during pedal operation is similar. The magnitude of the joint torque when the driver operates the three pedals at different seat heights is hip > knee > ankle.

In subsequent studies, we will consider the effects of more cab design parameters (steering wheels, gear sticks, etc.) on drivers’ musculoskeletal biomechanical characteristics and introduce the study of drivers’ lumbar spine and upper-body muscles.

## Figures and Tables

**Table 1:** Layout parameters of the driving simulator.

Parameters	Range or Value
seat backrest angle	8~18°
vertical h-point rise	0°
steering wheel inclination angle	30°
steering wheel diameter/mm	450
H-point heights (H30)/mm	405, 455, 505
pedal inclination angles (H30, A47)	(405, 45), (455, 36), (505, 27)
pedal center heights/mm (A47, Z_AP_)	(45, 141), (36, 117), (27, 91)

**Table 2:** Participants’ physical parameters.

Participants	Age	Height (cm)	Weight (kg)
1	24	176	78
2	25	178	82
3	26	178	78
Mean ± std	25 ± 1	177.33 ± 1.54	79.33 ± 2.3

**Table 3:** Participants’ physical parameters.

Parameters	Values (Participants 1 to 3)
leg length/cm	80, 83, 88
knee width/cm	10, 10.5, 18
ankle width/cm	10, 8, 10
elbow width/cm	9.5, 8.5, 8
wrist width/cm	6.5, 6.5, 7.5
palm thickness/cm	3.5, 3.5, 2.5
distance between the peak of the shoulder and the motion center of the shoulder joint/cm	15, 14.5, 8

## Data Availability

The data presented in this study are available on request from the corresponding author.
